# Age-Driven Cardiovascular Risk Scores Differ Across Antiretroviral Regimens Among People Living with HIV in the Colombian Caribbean Region

**DOI:** 10.3390/idr18050115

**Published:** 2026-10-07

**Authors:** Sthefanie Paola Parejo Garay, Juliana Andrea Gómez Perdomo, Eduardo Antonio Martínez Bula, Shirley Patricia Iglesias Pertuz, Sandra Paola Daguer Reyes, Alex Dominguez Vargas, Henry J. González-Torres

**Affiliations:** 1 Centro de Investigaciones en Ciencias de la Vida, Facultad de Ciencias de la Salud, Universidad Simón Bolívar, Barranquilla 080001, Colombia; sthefanie.parejo@unisimon.edu.co (S.P.P.G.); juliana.gomezp@unisimon.edu.co (J.A.G.P.); shirley.iglesias@unisimon.edu.co (S.P.I.P.); sandra.daguer@unisimon.edu.co (S.P.D.R.); 2 Departamento de Medicina Interna, Clínica de la Costa, Barranquilla 080001, Colombia; eduardo.martinezb@unisimon.edu.co; 3 Data Analysis and Mining Department, D&P Consulting Service SAS, Barranquilla 080001, Colombia; aadominguez17@hotmail.com

**Keywords:** HIV, cardiovascular risk, antiretroviral therapy, integrase strand-transfer inhibitors, Framingham, ASCVD, D:A:D, Colombia

## Abstract

Background/Objectives: People living with HIV (PLHIV) face an elevated burden of cardiovascular disease, and general-population and HIV-specific risk scores are commonly used for stratification, though data from Latin America, particularly Colombia’s Caribbean region, remain scarce. This study aimed to determine the distribution of cardiovascular risk, assessed through the Framingham, ASCVD ACC/AHA, and D:A:D scores, and to compare these scores across antiretroviral therapy (ART) regimens among PLHIV individuals in the Colombian Caribbean region. Methods: This cross-sectional study included 147 adult PLHIV patients receiving ART at care sites across the Colombian Caribbean. Sociodemographic, clinical, immunovirological, and treatment-related variables were compared across cardiovascular risk categories (low, moderate, high) using the Kruskal–Wallis test for continuous variables and Chi-squared or Fisher’s exact tests for categorical variables, as appropriate to expected cell counts. Agreement between the three scores was assessed with weighted Cohen’s kappa. Factors independently associated with high cardiovascular risk were evaluated using Bayesian multivariate logistic regression (full and parsimonious models). Results: All three cardiovascular risk scores differed significantly across ART regimen groups (*p* ≤ 0.005), with INSTI-containing regimens showing the highest scores. Agreement between scores was substantial between Framingham and ASCVD (κ = 0.717) and moderate between Framingham and D:A:D (κ = 0.534), and fair between ASCVD and D:A:D (κ = 0.321). Neither CD4+ count nor viral load status differed significantly by risk category. In multivariate analysis, age was the only variable independently associated with high cardiovascular risk (adjusted OR 1.34, 95% CI 1.18–1.53). Conclusions: Cardiovascular risk stratification in this Caribbean Colombian HIV cohort is driven primarily by chronological age; univariate differences by ART regimen group did not persist after formal comparison against an age-only model (likelihood-ratio *p* = 0.215), and are more consistent with channeling of older, higher-comorbidity patients toward INSTI-based regimens than with a direct pharmacological effect. Agreement between the three risk scores ranged from fair to substantial, indicating that scoring-system choice can meaningfully alter risk classification independently of virological or immunological control. These findings support age-focused cardiovascular monitoring in PLHIV individuals and caution against relying on a single, non-locally validated risk score in this population.

## 1. Introduction

Effective antiretroviral therapy (ART) has reshaped HIV into a manageable chronic illness, shifting much of its long-term clinical burden toward non-AIDS comorbidities, among which cardiovascular disease (CVD) now figures prominently. Relative to HIV-negative individuals, people living with HIV (PLHIV) face roughly double the risk of atherosclerotic events, a disparity that has widened considerably over the past twenty years [1]. Traditional risk factors alone do not account for this gap: ongoing immune activation, low-grade systemic inflammation stemming from the viral reservoir, gut microbial translocation, and chronic coinfections each independently accelerate the atherosclerotic process [2]. Much of what is known about cardiovascular risk distribution and prevention in PLHIV individuals, however, derives from European and North American cohorts, leaving considerable uncertainty about how well this evidence generalizes to settings with different risk-factor profiles and cardiovascular phenotypes [3].

Clinicians currently stratify cardiovascular risk in PLHIV individuals using instruments built for the general population—chiefly the Framingham and ASCVD ACC/AHA algorithms—alongside the HIV-specific Data-collection on Adverse Effects of Anti-HIV Drugs (D:A:D) score, which additionally factors in CD4 count and cumulative ART exposure [4]. Multinational evidence from the REPRIEVE trial, however, indicates that these general-population equations substantially mis-estimate cardiovascular events in PLHIV individuals, with the direction and magnitude of error varying by sex, race, and national income level, and calibration problems most pronounced in lower-income settings [5]. Echoing this, a systematic review of HIV-specific prediction models found no agreed-upon best-performing score and highlighted a near-absence of validation work from Latin America [6]. The only Colombian comparative data available come from one Bogotá-based academic hospital, studying a notably younger cohort (mean age: 35 years) skewed toward low and very-low risk, without any breakdown by ART regimen [7]. It therefore remains unknown whether cardiovascular risk scores vary meaningfully across current ART regimens within a Colombian population.

This evidence gap carries real clinical weight, since the specific drugs comprising a patient’s ART regimen can plausibly shift calculated cardiovascular risk. Integrase strand-transfer inhibitors (INSTIs)—now first-line therapy under most national guidelines, Colombia included—have repeatedly shown greater weight-gain liability than older drug classes across both trial and cohort data [8,9]. The multicentre RESPOND consortium extends this further, linking INSTI- and tenofovir alafenamide-related weight gain to a measurably higher incidence of hypertension and dyslipidaemia relative to other contemporary regimens [10]; both conditions feed directly into the Framingham, ASCVD, and D:A:D calculations. Whether this metabolic footprint actually translates into a detectable difference in composite cardiovascular risk category by ART regimen—particularly outside the high-income settings that generated most of this evidence—has not been tested. A key caveat, however, is that ART regimen assignment is not random: any regimen-linked risk difference observed could equally reflect clinicians preferentially channeling more cardio-metabolically vulnerable patients toward certain regimens, rather than a true drug effect.

Accordingly, this study aimed to determine the distribution of cardiovascular risk—assessed through the Framingham, ASCVD ACC/AHA, and D:A:D scores—and to compare these scores across antiretroviral therapy regimens among people living with HIV in the Colombian Caribbean region. Specific objectives were to describe the cohort’s sociodemographic and clinical profiles by cardiovascular risk category; to characterize immunobiological and virological status and its relationship to risk stratification; to describe ART regimen and care-modality patterns by risk category; to compare the three cardiovascular risk scores across regimen groups and quantify their concordance; and to identify independent correlates of high cardiovascular risk via multivariate logistic regression.

## 2. Materials and Methods

### 2.1. Study Design

We conducted an observational, cross-sectional, analytical study on people living with HIV (PLHIV) receiving antiretroviral therapy (ART) at referral centers across the Colombian Caribbean region. The primary objective was to determine the distribution of cardiovascular risk, assessed through three validated risk scores, and to compare these scores across ART regimen groups.

### 2.2. Study Population

The cohort comprised adult patients living with HIV enrolled in an institutional ART-care program operating at outpatient HIV-care sites in the departments of Atlántico, Cesar, Magdalena, Córdoba, and other associated sites within the Colombian Caribbean region since January 2024. For this cross-sectional study, each patient’s most recent clinical encounter recorded between January 2024 and 30 June 2025 was used for cardiovascular risk assessment. Inclusion criteria were age ≥ 18 years, confirmed HIV diagnosis, and calculable D:A:D cardiovascular risk score (the primary outcome measure). Framingham and ASCVD ACC/AHA scores were additionally calculated where clinically applicable—Framingham within its validated age range (30–74 years) and ASCVD ACC/AHA within its validated age range (20–79 years); scores not calculable due to these algorithm-specific age limits, rather than missing source data, account for the smaller denominators reported for these two scores in Section 3. Exclusion criteria were incomplete source data precluding D:A:D calculation and prior diagnosis of cardiovascular disease. After data cleaning and verification of source variables, a total of 147 patients were included in the final analysis.

### 2.3. Definitions and Variables

Sociodemographic variables (age, sex, site of care), clinical variables (hypertension, type 2 diabetes mellitus, smoking status, hypercholesterolemia, hypertriglyceridemia, mixed hyperlipidemia), immunobiological and virological variables (CD4+ T-cell count, HIV viral load, WHO/CDC clinical stage, PPD skin test result, syphilis screening, and Pneumocystis jirovecii pneumonia prophylaxis status), and treatment-related variables (ART regimen group, time since ART initiation, care modality) were collected. Cardiovascular risk category was defined as low, moderate, or high according to the cut-off points established for each of the three risk scores (see Section 2.5).

### 2.4. Data Collection

Information was extracted from institutional electronic health records and HIV-care program registries at each participating site. Framingham and ASCVD ACC/AHA scores were recalculated from source clinical and laboratory variables (systolic blood pressure, total and HDL cholesterol, antihypertensive treatment, smoking status, diabetes status, age, sex, and race) using the D’Agostino et al. [11] and ACC/AHA (2013) [12] pooled cohort equation coefficients, respectively, after an initial calculation discrepancy between the two scores was identified during data cleaning. The D:A:D score was retained as originally calculated and verified for internal consistency—specifically, its expected positive association with age—rather than recalculated de novo, as cumulative ART-exposure-years data required for full recalculation were not available in reusable form. ART regimen group was determined from individual medication-dispensing records rather than a single aggregated regimen field: for each patient, the antiretroviral drug classes present across all dispensed products—integrase strand-transfer inhibitor [INSTI], nucleos(t)ide reverse-transcriptase inhibitor [NRTI], non-nucleoside reverse-transcriptase inhibitor [NNRTI], and protease inhibitor [PI]—were identified from recorded active-ingredient data and combined into one of five mutually exclusive categories (PI, NNRTI + NRTI, NRTI + INSTI, INSTI, NRTI), with PI- and NNRTI-containing combinations classified by anchor agent regardless of backbone composition.

### 2.5. Cardiovascular Risk Score Calculation

Cardiovascular risk was estimated using three validated instruments: the Framingham risk score, the Atherosclerotic Cardiovascular Disease (ASCVD) risk score of the American College of Cardiology/American Heart Association, and the Data-collection on Adverse Effects of Anti-HIV Drugs (D:A:D) score, the latter developed specifically for PLHIV. Each score was categorized into low, moderate, or high cardiovascular risk according to its respective established thresholds: Framingham risk < 10%, 10–<20%, and ≥20%; ASCVD risk < 5%, 5–<20%, and ≥20%; and D:A:D 10-year risk < 1%, 1–5%, and >5%, respectively.

### 2.6. Classification of Antiretroviral Therapy Regimens

ART regimens were grouped into five mutually exclusive categories based on drug class composition: integrase strand-transfer inhibitors (INSTI), nucleoside/nucleotide reverse transcriptase inhibitors plus integrase inhibitors (NRTI + INSTI), NRTI-based regimens, non-nucleoside reverse transcriptase inhibitors plus NRTI (NNRTI + NRTI), and protease inhibitor (PI)-based regimens. Because the initially recorded aggregate regimen field showed internal inconsistencies on cross-checking against individual medication records, classification was instead derived from patient-level medication-dispensing data: for each patient, the antiretroviral drug classes present across all dispensed products were identified from recorded active-ingredient and dose information and combined into one of the five categories above, with PI- and NNRTI-containing combinations classified by anchor agent regardless of backbone composition.

### 2.7. Statistical Analysis

Normality of quantitative variables was assessed with the Kolmogorov–Smirnov test. Continuous variables were described as medians and interquartile ranges (Q1–Q3), and categorical variables as absolute and relative frequencies. Between-group comparisons across cardiovascular risk categories (low, moderate, high) were performed using the Kruskal–Wallis rank-sum test for continuous variables and Pearson’s Chi-squared or Fisher’s exact test for categorical variables, as appropriate to expected cell counts. Pairwise post hoc comparisons used the Wilcoxon rank-sum test with Holm correction for multiple comparisons.

Factors independently associated with the high cardiovascular risk category were identified through Bayesian logistic regression (bayesglm, weakly informative Cauchy priors), reported as odds ratios (OR) with 95% Wald-type confidence intervals. Given the limited number of high-risk events (*n* = 22) relative to the number of candidate predictors, a complete model (13 parameters, events-per-variable [EPV] = 1.69) was fitted as an exploratory analysis, and a parsimonious model (4 parameters, EPV = 5.50) was fitted as the primary inferential model. A *p*-value < 0.05 was considered statistically significant. All analyses were conducted using R statistical software (version 4.5.3; R Foundation for Statistical Computing, Vienna, Austria).

### 2.8. Ethical Considerations

The study was conducted in accordance with the ethical principles of the Declaration of Helsinki and Colombian national health research regulations (Resolution 8430 of 1993, Ministry of Health). Confidentiality was ensured by anonymizing data, with no access to personal identifiers. As this was a retrospective, non-interventional study, additional informed consent was not required. The protocol was reviewed and approved by the Scientific Committee of the Faculty of Health Sciences at Universidad Simón Bolívar through the act 640-027-2026 (13 May 2026) and by the Research Ethics Committee of Clínica de la Costa in Act 566 (29 July 2026).

## 3. Results

### 3.1. Cohort Characteristics

A total of 147 patients living with HIV on antiretroviral therapy (ART) were included in the final corrected analysis. Median age was 48.0 years (IQR 43.0–55.0), and 129 patients (88%) were male. Hypertension (25.2%), type 2 diabetes (7.5%), current smoking (8.8%), hypercholesterolemia (16.3%), and mixed hyperlipidemia (8.8%) all increased significantly across cardiovascular risk categories (*p* = 0.001, *p* = 0.014, *p* < 0.001, *p* = 0.007, and *p* = 0.004, respectively), while hypertriglyceridemia (23.1%, *p* = 0.084) showed a non-significant trend. Geographic distribution across Caribbean departments did not differ uniformly by risk category: patients from Atlántico were significantly overrepresented among moderate/high-risk categories (*p* = 0.002), while patients from Cesar were significantly overrepresented among low-risk patients (*p* = 0.002); no other department showed a significant association with risk category (all *p* ≥ 0.09) (Table 1).

Age increased progressively and significantly across the three D:A:D risk categories (Low: 43.0 [42.0–45.0]; Moderate: 49.0 [44.0–54.0]; High: 59.0 [58.0–62.0] years; *p* < 0.001 for all pairwise comparisons). Median CD4+ T-cell count was 508.0 cells/mm^3^ (IQR 347.0–724.5) and did not differ significantly across cardiovascular risk categories (*p* = 0.25), nor did HIV clinical stage (*p* = 0.96) or detectable viral load status (*p* = 0.42) (Table 1). Viral load was numerically higher in the high-risk category (6263 copies/mL [IQR 1610–171,000]) than in the moderate- (289 [100–3575]) or low-risk groups (1187 [88–12,453]) among patients with detectable viremia, although this difference did not reach statistical significance (*p* = 0.21).

### 3.2. Antiretroviral Therapy Characteristics

There was no significant difference in time since ART initiation (*p* = 0.54) or care modality (in-person vs. teleconsultation, *p* = 0.41) across cardiovascular risk categories. Regimen composition, however, differed significantly: use of NRTI + INSTI-based regimens increased markedly with risk category (30% in low risk vs. 82% in high risk; *p* < 0.001), while PI-based regimens showed a significant inverse trend (41% to 14%; *p* = 0.043), and NNRTI + NRTI regimens showed a borderline inverse trend (23% to 5%; *p* = 0.082) (Table 2).

**Figure 1:**
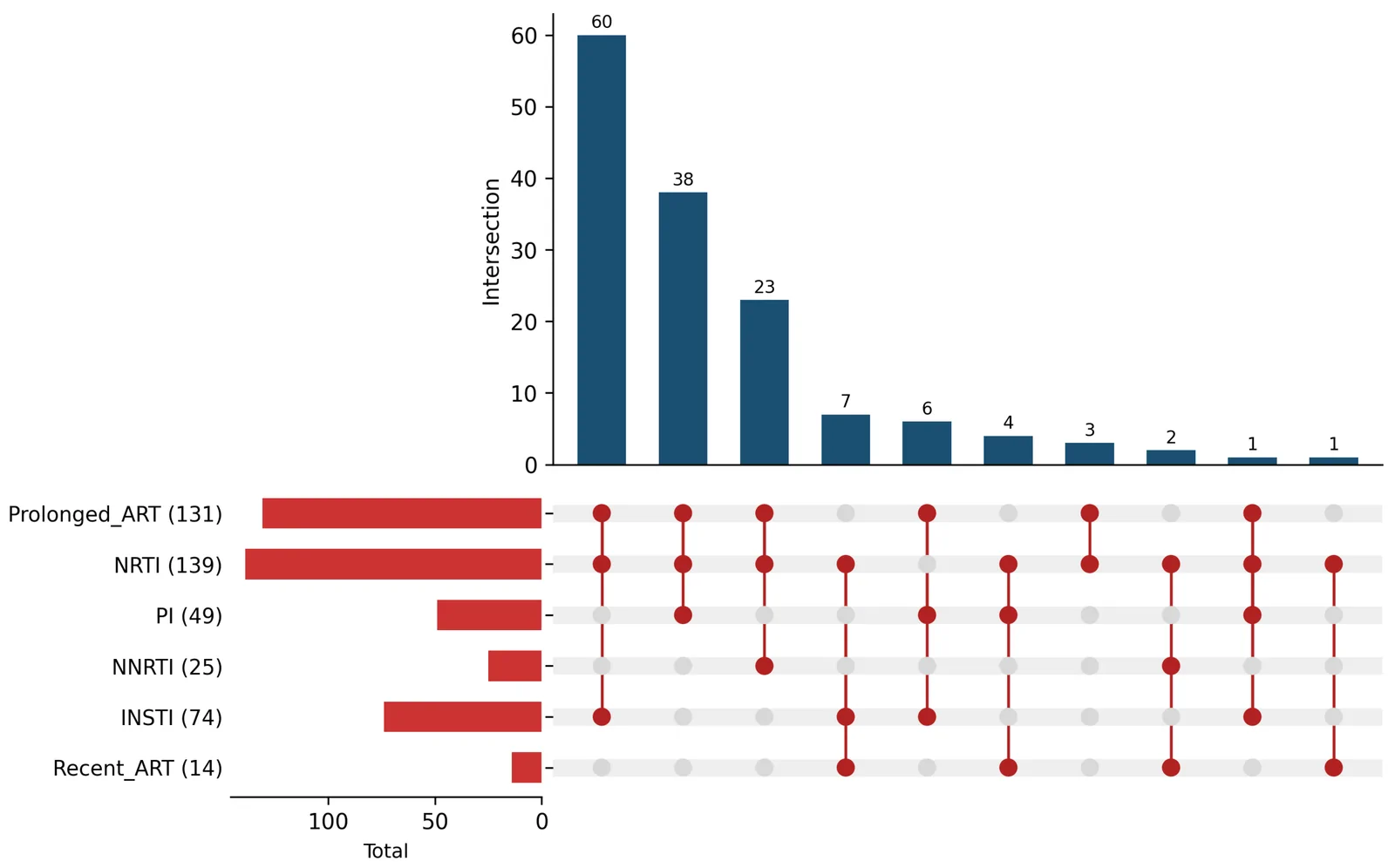
UpSet plot showing the distribution and overlap of antiretroviral therapy (ART) drug-class combinations and treatment-duration status across the cohort (*n* = 145; two patients with no classifiable ART regimen were excluded). Blue vertical bars show the number of patients with each combination; filled red dots connected by vertical lines indicate the drug classes and treatment-duration status that define each combination; red horizontal bars (left) show the total number of patients in each category. Abbreviations: ART, antiretroviral therapy; INSTI, integrase strand-transfer inhibitor; NRTI, nucleoside/nucleotide reverse transcriptase inhibitor; NNRTI, non-nucleoside reverse transcriptase inhibitor; PI, protease inhibitor. Prolonged_ART and Recent_ART indicate ART initiation ≥ 48 weeks and <48 weeks before the assessment date, respectively.

ART drug-class combinations varied substantially across the cohort, with INSTI + NRTI regimens used as prolonged therapy being the most frequent combination (*n* = 60), followed by NRTI + PI (*n* = 38) and NNRTI + NRTI (*n* = 23), both also predominantly used as prolonged therapy (Figure 1).

### 3.3. Cardiovascular Risk Scores Across ART Regimen Groups

All three cardiovascular risk scores differed significantly across ART regimen groups (Kruskal–Wallis, ASCVD *p* = 0.001, Framingham *p* = 0.005, D:A:D *p* = 0.002). Median D:A:D score was highest among patients on NRTI + INSTI regimens (2.2%, IQR 1.1–5.2), followed by NNRTI + NRTI (1.4%, IQR 0.8–2.2) and PI-based regimens (1.2%, IQR 0.7–2.5); the NRTI-only group showed the lowest median (0.8%) but comprised only 4 patients and should be interpreted with caution, with the same ranking observed for the ASCVD and Framingham scores (Table 3). Despite these regimen-related differences, the three scores were strongly and significantly correlated with one another (Spearman’s ρ = 0.95–0.97, all *p* < 0.001), with agreement on categorical risk classification ranging from fair (ASCVD vs. D:A:D, weighted κ = 0.32) to substantial (Framingham vs. ASCVD, weighted κ = 0.72).

**Figure 2:**
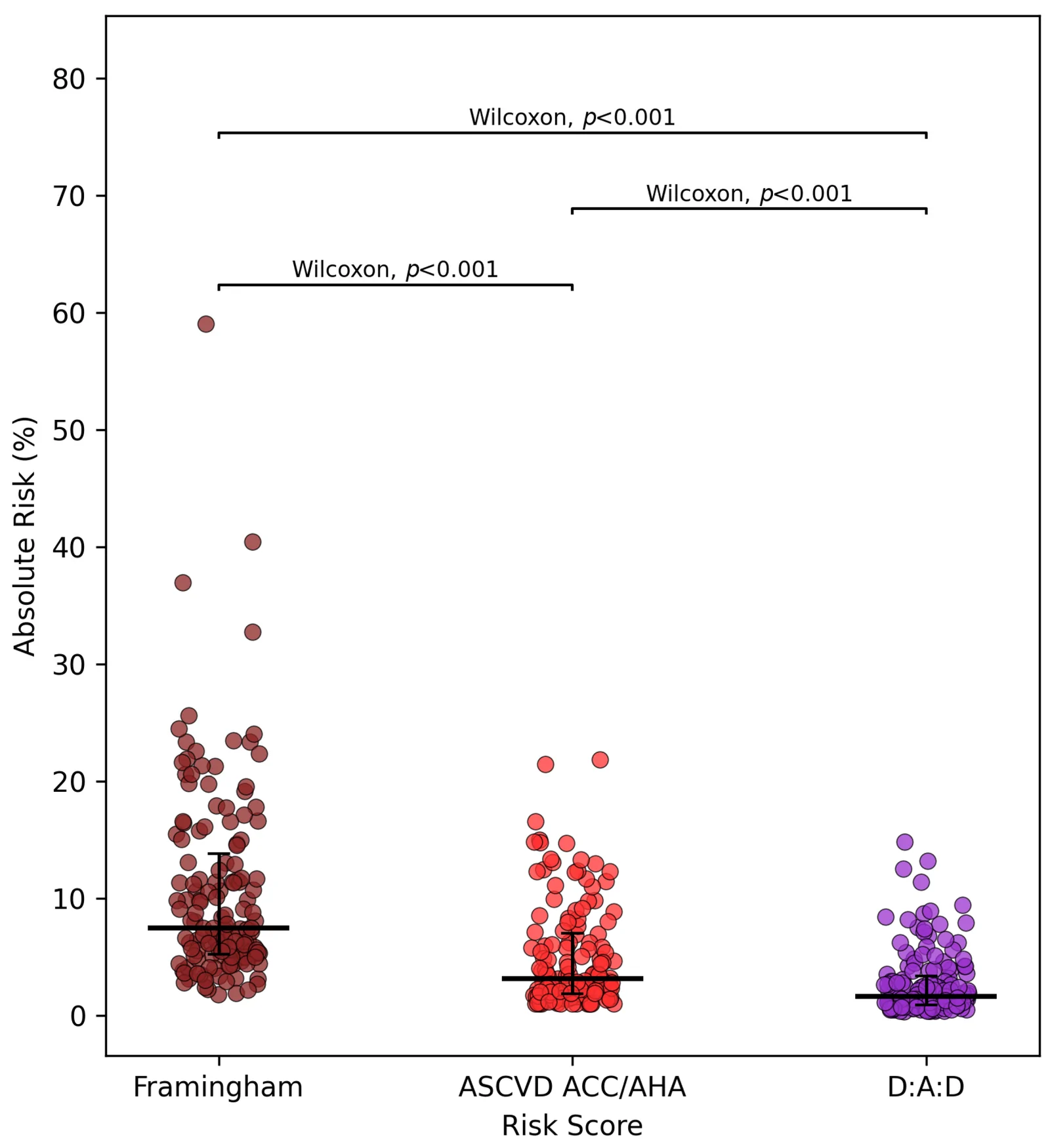
Comparison of absolute cardiovascular risk estimates across the Framingham, ASCVD ACC/AHA, and D:A:D scoring systems within the same patients. Horizontal bars represent the median, with error bars indicating the interquartile range (Q1–Q3). Statistical comparisons were performed using the Wilcoxon rank-sum test with Holm correction for multiple comparisons.

Comparing the three scoring systems directly within the same patients, Framingham consistently yielded the highest absolute risk estimates, followed by ASCVD ACC/AHA, with D:A:D producing the lowest estimates (all pairwise comparisons *p* < 0.001, Holm-corrected) (Figure 2). Agreement between scoring systems, assessed with weighted Cohen’s kappa, was substantial between Framingham and ASCVD (κ = 0.717, *n* = 136, *p* < 0.001) and moderate between Framingham and D:A:D (κ = 0.534, *n* = 147, *p* < 0.001), but only fair between ASCVD and D:A:D (κ = 0.321, *n* = 136, *p* < 0.001)—indicating that the two general-population-derived scores (Framingham, ASCVD) classify patients more similarly to each other than either does to the HIV-specific D:A:D score.

### 3.4. Factors Associated with High Cardiovascular Risk

Given the limited number of high-risk events relative to the number of candidate predictors in the full model (22 high-risk cases; 13 parameters; events per variable [EPV] = 1.69), this model was treated as exploratory. In the full model, age was the only variable independently associated with high cardiovascular risk (adjusted OR 1.34, 95% CI 1.18–1.53, *p* < 0.001); no other covariate—including sex, site, HIV clinical stage, viral load status, time since ART initiation, or ART regimen group—reached statistical significance (Table 4).

A parsimonious model restricted to age and ART regimen group (4 parameters; EPV = 5.50) was fitted as the primary inferential model. Age remained the only independent predictor of high cardiovascular risk (adjusted OR 1.29, 95% CI 1.16–1.45, *p* < 0.001); none of the ART regimen groups differed significantly from the NRTI + INSTI reference category. Both models showed excellent discrimination (AUC > 0.90 per standard classification); the parsimonious model retained comparable discrimination (AUC 0.945 full vs. 0.929 parsimonious) with markedly improved parameter efficiency (EPV 5.50 vs. 1.69), supporting its use as the primary inferential model (Table 4).

## 4. Discussion

In this Caribbean Colombian cohort of people living with HIV, chronological age emerged as the dominant, and in the multivariate analysis the only independent, correlate of high cardiovascular risk. Although patients on NRTI + INSTI-containing regimens showed higher unadjusted risk scores than those on other regimens, this association did not survive formal comparison against an age-only model (likelihood-ratio *p* = 0.215), indicating that regimen distribution in this cohort is better explained by older, more comorbid patients being preferentially maintained on these regimens than by a direct pharmacological effect. The three risk-scoring instruments themselves showed only fair-to-substantial pairwise agreement, with the weakest concordance specifically between the general-population-derived ASCVD score and the HIV-specific D:A:D score. Neither CD4+ count nor viral load status differed meaningfully across risk categories.

Placed against prior comparative work, this agreement pattern is partly consistent with, and partly distinct from, earlier reports. Souza and colleagues documented near-perfect concordance between D:A:Dr and Framingham in a Brazilian cohort (κ = 0.82), alongside substantial agreement of both scores against ASCVD (κ = 0.70–0.74) [13]. The UK POPPY study similarly reported moderate-to-substantial Framingham–D:A:D agreement (κ = 0.51–0.65) in a largely white population [14].

Our Framingham–ASCVD concordance (κ = 0.717) falls squarely within this range, and even exceeds it when benchmarked against a rural South African cohort, where Framingham and D:A:D agreed only fairly (κ = 0.23) [15], or an Argentine–Uruguayan analysis reporting substantially divergent high-risk cut-points across cardiovascular formulas [16]. What distinguishes our findings is not a uniformly weaker concordance typical of under-resourced settings, but a specific fault line: the two general-population instruments agreed with each other about as well as, or better than, in the Brazilian and UK cohorts, while the HIV-specific D:A:D score diverged more sharply from both—most markedly from ASCVD (κ = 0.321). Discordance here appears concentrated at the general-population/HIV-specific interface rather than spread evenly across all three instruments, precisely the pairing for which population-specific calibration would matter most.

This carries a concrete clinical consequence. Durstenfeld and colleagues, comparing the newer PREVENT equations against the pooled cohort equations in a US sample, found systematic differences in predicted risk by race and sex, with PREVENT recommending statin therapy to under half as many patients at a shared threshold [17]. Grand et al. reached a related conclusion using a carotid-plaque surrogate, showing that every equation tested, D:A:D included, underestimated true cardiovascular risk, with guideline-recommended cut-points poorly matched to locally optimal ones [16]. Our own finding—that simply switching which score is applied can reclassify a Caribbean Colombian patient into a different risk tier—reinforces the same conclusion: absent locally calibrated thresholds, basing statin-initiation or referral decisions on any single imported instrument risks misclassifying patients in either direction.

The lack of association between CD4+ count, viral load, and risk category aligns with recent work on subclinical atherosclerosis. Farrant et al. found that CD4 count, viral load, and ART regimen were unrelated to carotid intima-media thickness in a Kenyan cohort, whereas blood pressure was [18]; their outcome was a direct imaging measure, while our composite scores already embed blood pressure, lipids, and age by construction, so the parallel here applies specifically to the CD4/viral-load null finding rather than to ART regimen, which—unlike in their study—did show a univariate, if not multivariate, association with risk category in our cohort (Table 2 and Table 3). Blaauw et al. reported a similar pattern in a large European cohort, where conventional risk factors, not HIV-specific immunovirological markers, best explained intima-media thickness [19]. Our results extend this logic from imaging markers to composite risk scores: once a patient is established on ART, cardiovascular risk in this cohort appears governed largely by conventional, age- and metabolism-linked pathways rather than by residual immunodeficiency or viremia.

The unadjusted association between NRTI + INSTI regimens and higher composite scores is biologically plausible in principle. Using target trial emulation within REPRIEVE, Kileel et al. found that switching to an INSTI-based regimen raised five-year incidence of obesity, diabetes, and hypertension, though not of major adverse cardiovascular events over that horizon [20]; a parallel North American AIDS Cohort Collaboration analysis reported a 38% higher incident-diabetes risk after a protease-inhibitor-to-INSTI switch [21]. Since obesity, diabetes, and hypertension enter the Framingham, ASCVD, and D:A:D algorithms directly, a mechanistic INSTI effect of this kind would be expected to raise composite scores even before any difference in hard events appears. In our data, however, this regimen association did not persist once age was accounted for, and age itself did not differ significantly between regimen groups (*p* = 0.20), arguing against the simplest version of confounding through age imbalance. The likelihood-ratio comparison instead suggests that age alone captures essentially all the discriminative signal the fuller model offered. Taken together with the cross-sectional, non-randomized nature of regimen assignment, the most defensible reading of our data is that older patients with greater baseline cardiometabolic burden were preferentially maintained on NRTI + INSTI regimens, a channeling phenomenon, rather than that the regimen itself drove the observed score elevation. This does not contradict the mechanistic evidence from Kileel and Hwang; it simply means our cross-sectional design, with a comparatively small high-risk stratum, was not positioned to detect a regimen effect independent of age, even if one exists.

To our knowledge, this is the first study to characterize cardiovascular risk scores by ART regimen specifically within Colombia’s Caribbean region. Colombia’s only prior comparative data come from a single Bogotá hospital, studying a younger, lower-risk cohort without regimen stratification [7]. Given how much the Caribbean coast differs from the Andean interior sociodemographically, epidemiologically, and in health-system structure, this was a genuine blind spot, and our results offer the first age- and regimen-stratified reference point for clinicians managing PLHIV along Colombia’s coast.

Several limitations temper these conclusions. The full multivariate model rested on few high-risk events (22) relative to its parameter count, yielding an events-per-variable ratio below conventional thresholds [22,23]; Bayesian regularization mitigates but does not eliminate this instability, so even the better-powered parsimonious model should not be read as establishing causal regimen effects. Because ART regimen was not randomly assigned, confounding by indication remains a live possibility, and continuous ART duration was unavailable—only a binary <48/≥48-week cutoff—though age did not differ significantly between regimen groups (*p* = 0.20), arguing against the simplest version of this confound. Age’s outsized contribution to model discrimination is partly mechanical, since age enters the D:A:D score’s own formula directly rather than acting purely as an external predictor. Finally, the denominators underlying our kappa estimates varied (136–147 of 147 patients) because Framingham and ASCVD are only defined within fixed age windows (30–74 and 20–79 years, respectively)—an algorithmic constraint, not missing data or cohort attrition.

## 5. Conclusions

Cardiovascular risk in this Caribbean Colombian cohort of people living with HIV is driven primarily by chronological age, while the scoring system applied can independently shift a patient’s risk classification, independently of virological or immunological control. Regimen-associated differences in unadjusted analyses did not persist after accounting for age, and are more consistent with channeling of older, higher-comorbidity patients toward specific regimens than with a direct pharmacological effect; these findings support age-focused metabolic monitoring in routine follow-up for people living with HIV more broadly, consistent with emerging global trial evidence on the metabolic effects of ART regimen switches, and argue against relying on any single imported risk score without local validation in this population. Prospective, longitudinal studies with time-updated ART exposure and hard cardiovascular endpoints are needed to determine whether age-related and regimen-related score differences observed here translate into differences in actual cardiovascular events in this specific population.

## Figures and Tables

**Table 1:** Baseline sociodemographic, clinical, immunobiological, and treatment characteristics by D:A:D cardiovascular risk category.

Characteristic	Low *n* = 44 ^1^	Moderate *n* = 80 ^1^	High *n* = 23 ^1^	*p*-Value
Age	43 [42–45]	49 [44–54]	59 [58–62]	<0.001 ^2^
Sex				0.7 ^3^
Female	7 (16%)	9 (11%)	2 (8.7%)	
Male	37 (84%)	71 (89%)	21 (91%)	
Hypertension	2 (4.5%)	27 (34%)	8 (35%)	<0.001 ^4^
Type 2 diabetes mellitus	0 (0%)	7 (8.8%)	4 (17%)	0.014 ^3^
Current smoker	0 (0%)	6 (7.5%)	7 (30%)	<0.001 ^3^
Hypercholesterolemia	2 (4.5%)	14 (18%)	8 (35%)	0.007 ^3^
Hypertriglyceridemia	5 (11%)	23 (29%)	6 (26%)	0.084 ^4^
Mixed hyperlipidemia	0 (0%)	8 (10%)	5 (22%)	0.004 ^3^
Site of care, *n* (%)				0.005 ^3^
Atlántico	4 (9.1%)	30 (38%)	9 (39%)	
Cesar	17 (39%)	9 (11%)	3 (13%)	
Magdalena	4 (9.1%)	9 (11%)	2 (8.7%)	
Montería	13 (30%)	23 (29%)	7 (30%)	
Sincelejo	5 (11%)	6 (7.5%)	0 (0%)	
Other sites (*n* = 3)	1 (2.3%)	3 (3.8%)	2 (8.7%)	
CD4+ count (cells/mm^3^)	504 [327–745]	499 [337–670]	583 [470–774]	0.25 ^2^
Clinical stage				0.96 ^3^
I	22 (50%)	39 (49%)	13 (57%)	
II	20 (45%)	36 (45%)	9 (39%)	
III	2 (4.5%)	5 (6.3%)	1 (4.3%)	
Viral load, Detectable	12 (27%)	18 (23%)	3 (13%)	0.42 ^4^
Syphilis, Positive	17 (39%)	13 (16%)	6 (26%)	0.021 ^4^
Among patients with detectable viremia	*n* = 12	*n* = 18	*n* = 3	
Viral load (copies/mL)	1187 [88–12,453]	289 [100–3575]	6263 [1610–171,000]	0.21 ^2^

^1^ Median [Q1–Q3]; *n* (%); ^2^ Kruskal–Wallis rank sum test; ^3^ Fisher’s exact test; ^4^ Pearson’s Chi-squared test.

**Table 2:** Antiretroviral therapy regimen and care modality characteristics by cardiovascular risk category.

Characteristic	Low *n* = 44 ^1^	Moderate *n* = 80 ^1^	High *n* = 23 ^1^	*p*-Value
Time since ART initiation				0.54 ^2^
<48 weeks	6 (14%)	6 (7.5%)	2 (8.7%)	
≥48 weeks	38 (86%)	74 (93%)	21 (91%)	
Care modality				0.41 ^2^
In-person	41 (93%)	77 (96%)	23 (100%)	
Teleconsultation	3 (6.8%)	3 (3.8%)	0 (0%)	
ART regimen group ^3^	*n* = 44	*n* = 79	*n* = 22	
NRTI + INSTI	13 (30%)	36 (46%)	18 (82%)	<0.001 ^4^
PI	18 (41%)	28 (35%)	3 (14%)	0.043 ^4^
NNRTI + NRTI	10 (23%)	14 (18%)	1 (4.5%)	0.082 ^4^
NRTI alone	3 (6.8%)	1 (1.3%)	0 (0%)	0.065 ^4^

^1^ *n* (%); ^2^ Fisher’s exact test; ^3^ 2 patients with no classifiable ART regimen excluded (Materials and Methods Section 2.6); ^4^ Cochran–Armitage trend test across ordered risk categories, per regimen group. “NRTI alone” = NRTI backbone with no accompanying INSTI, NNRTI, or PI (Materials and Methods Section 2.6). Abbreviations: ART, antiretroviral therapy; INSTI, integrase strand-transfer inhibitor; NRTI, nucleoside/nucleotide reverse transcriptase inhibitor; NNRTI, non-nucleoside reverse transcriptase inhibitor; PI, protease inhibitor.

**Table 3:** Predictive cardiovascular risk scores by antiretroviral therapy regimen group.

Characteristic	NRTI + INSTI *n* = 67 ^1^	PI *n* = 49 ^1^	NNRTI + NRTI *n* = 25 ^1^	NRTI *n* = 4 ^1^	*p*-Value
ASCVD score (%)	4.8 [2.5–9.9]	2.2 [1.5–4.6]	2.7 [1.8–4.8]	1.8 [1.3–2.6]	0.001 ^2^
ASCVD risk category	*n* = 59	*n* = 47	*n* = 24	*n* = 4	0.011 ^3^
Low	30 (51%)	37 (79%)	18 (75%)	4 (100%)	
Moderate	27 (46%)	10 (21%)	6 (25%)	0 (0%)	
High	2 (3.4%)	0 (0%)	0 (0%)	0 (0%)	
Framingham score (%)	10.7 [5.7–19.3]	6.2 [4.5–9.9]	7.5 [5.1–9.7]	5.3 [4.3–7.1]	0.005 ^2^
Framingham risk category					0.006 ^3^
Low	31 (46%)	37 (76%)	19 (76%)	4 (100%)	
Moderate	22 (33%)	9 (18%)	5 (20%)	0 (0%)	
High	14 (21%)	3 (6.1%)	1 (4.0%)	0 (0%)	
D:A:D score (%)	2.2 [1.1–5.2]	1.2 [0.7–2.5]	1.4 [0.8–2.2]	0.8 [0.6–1.1]	0.002 ^2^
D:A:D risk category					0.004 ^3^
Low	13 (19%)	18 (37%)	10 (40%)	3 (75%)	
Moderate	36 (54%)	28 (57%)	14 (56%)	1 (25%)	
High	18 (27%)	3 (6.1%)	1 (4.0%)	0 (0%)	

^1^ Median [Q1–Q3]; *n* (%); ^2^ Kruskal–Wallis rank sum test; ^3^ Fisher’s exact test. Abbreviations: ASCVD, Atherosclerotic Cardiovascular Disease risk score; D:A:D, Data collection on Adverse effects of anti-HIV Drugs; INSTI, integrase strand-transfer inhibitor; NRTI, nucleoside/nucleotide reverse transcriptase inhibitor; NNRTI, non-nucleoside reverse transcriptase inhibitor; PI, protease inhibitor.

**Table 4:** Factors associated with high cardiovascular risk—Bayesian multivariate logistic regression, full and parsimonious models (N = 145; 22 high-risk cases; 2 patients with no classifiable ART regimen excluded).

Characteristic	Full model ^1^ aOR (95% CI)	*p*	Parsimonious Model ^2^ aOR (95% CI)	*p*
Age	1.34 (1.18–1.53)	<0.001	1.29 (1.16–1.45)	<0.001
Sex: Male	2.37 (0.43–13.01)	0.319	—	—
Site:				
Cesar	2.23 (0.33–15.00)	0.411	—	—
Magdalena	2.54 (0.36–17.76)	0.348	—	—
Montería	1.71 (0.39–7.45)	0.473	—	—
Other ^3^	1.20 (0.14–10.56)	0.867	—	—
Clinical stage				
II	0.45 (0.12–1.66)	0.229	—	—
III	0.54 (0.04–6.95)	0.640	—	—
Viral load: Undetectable	1.14 (0.24–5.48)	0.873	—	—
Time since ART initiation ≥ 48 weeks	0.66 (0.07–6.19)	0.716	—	—
ART regimen group				
PI	0.25 (0.05–1.24)	0.090	0.35 (0.09–1.41)	0.141
NNRTI + NRTI	0.19 (0.02–1.71)	0.139	0.30 (0.04–2.05)	0.220
NRTI	0.78 (0.03–18.76)	0.880	0.77 (0.03–18.16)	0.873
Parameters/EPV/AUC	13/1.69/0.945		4/5.50/0.929	

^1^ Reference categories: female, Atlántico, stage I, detectable viral load, ART initiation < 48 weeks, NRTI + INSTI regimen; Bayesian logistic regression, weakly informative Cauchy priors, Wald-type 95% CI. ^2^ Restricted to age and ART regimen group (reference: NRTI + INSTI), same estimation method. ^3^ “Other” here includes Sincelejo in addition to the departments grouped as “Other” in Table 1 (Cartagena, La Guajira, San Andrés), due to further cell-size constraints in the multivariate model. Dashes (—) indicate the reference category (fixed at OR = 1.00, no CI/*p*-value) for site, stage and ART regimen group, or a covariate not included in the parsimonious model. Abbreviations: OR, adjusted odds ratio; CI, confidence interval; ART, antiretroviral therapy; INSTI, integrase strand-transfer inhibitor; NRTI, nucleoside/nucleotide reverse transcriptase inhibitor; NNRTI, non-nucleoside reverse transcriptase inhibitor; PI, protease inhibitor; EPV, events per variable.

## Data Availability

The data presented in this study are available within this article. The anonymized data underlying the results can also be made available upon reasonable written request to the corresponding authors, accompanied by a justification for its use.

## Abbreviations

The following abbreviations are used in this manuscript:

ACC/AHA	American College of Cardiology/American Heart Association
aOR	Adjusted odds ratio
ART	Antiretroviral therapy
ASCVD	Atherosclerotic Cardiovascular Disease
AUC	Area under the curve
CD4	Cluster of differentiation 4
CI	Confidence interval
CVD	Cardiovascular disease
D:A:D	Data-collection on Adverse Effects of Anti-HIV Drugs
EPV	Events per variable
HIV	Human immunodeficiency virus
INSTI	Integrase strand-transfer inhibitor
IQR	Interquartile range
MACE	Major adverse cardiovascular events
NNRTI	Non-nucleoside reverse transcriptase inhibitor
NRTI	Nucleoside/nucleotide reverse transcriptase inhibitor
OR	Odds ratio
PI	Protease inhibitor
PLHIV	People living with HIV
PPD	Purified protein derivative (tuberculin skin test)
REPRIEVE	Randomized Trial to Prevent Vascular Events in HIV
